# Psychiatry

**Published:** 1905-07-01

**Authors:** 


### Progress in Medicine and Surgery.

### PSYCHIATRY.

**General Paralysis Following Syphilis in Wives.**—General paralysis in married women, arising after syphilitic infection from the husband, is a rare occurrence, but sufficiently numerous cases have at present been investigated to afford support to the theory that conjugal syphilis (contracted by one from the other partner in married life) is a most potent cause of general paralysis. The subject was first dealt with by Ludwig Acker (1887) and Mendel (1888), both of whom reported cases of general paralysis caused in wives by syphilis contracted from their husbands. Various observers have since then placed similar cases on record, as at present, according to Cullere[1] (1904) no fewer than 40 undoubted cases are known to medical literature. In a recent publication,[2] Drs. S. Garnier and A. Santenoise of the Dijon Asylum, give an interesting account of a case of "conjugal general paralysis." The patient was an old woman with a neuropathic family history, her mother having been affected with insane delusions, while a first cousin, on the mother's side, had epilepsy and in adult life developed into a general paralytic of the expansive or grandiose type. The patient married at 49 years of age, but was separated a year later from her husband, who was then taken to an asylum suffering from general paralysis, and who a few days after admission died of "apoplectiform" attacks. (His father had been a patient in the asylum previously, and had died from cerebral softening and organic dementia, while an uncle was a senile dement, with intercurrent maniacal attacks). Shortly after her marriage (1896) she presented signs of syphilitic infection, namely, roseolous rash, sore throat, and other troubles, and was diagnosed as a case of recent syphilis by Dr. P—— of Dijon. She, however, refused treatment, saying "she would not be poisoned with medicines." Eight years later she developed all the typical signs of general paralysis—speech embarrassment, tremulous articulation, inequality and contraction of the pupils, tremors of the tongue and lips, difficulty of locomotion, enfeeblement of memory, dementia and untidy and dirty habits. Paroxysmal apoplectiform attacks ensued, and she died in the course of one of these attacks. The interval between syphilitic infection, and the establishment of general paralysis in this case, was about eight years.

**Tanzi's Classification of Insanity.**—Professor Tanzi, of Florence, a distinguished alienist physician,[3] and superintendent of the asylum at Milan has recently published an excellent classification of the insanities, which is as follows. The basis of the classification is etiological and to some extent symptomatological. The following eight "groups" of insanity are recognised:—

(a). *Infantile cerebropathies.*
1. Idiocy.
2. Imbecility.

(b). *Constitutional neuro-psychoses.*
3. Constitutional neurasthenic insanity.
4. Hysterical insanity.
5. Epileptic insanity.

(c). *Degenerative anomalies of mind.*
6. Perversion of the sexual instinct.
7. Constitutional immorality.
8. Hereditary weak-mindedness.
9. Paranoia.

(d). *Dementia præcox.*

10. Hebephrenia.

11. Catatonia.

12. Paranoid forms of d. præcox.

e). *Affective psychoses.*
13. Mania.
14. Melancholia.
15. Circular insanity.

(f). *Toxic insanity.*
16. Alcoholic insanity.
17. Morphinism, cocainism.
18. Pellagra.

(g). *Insanity of infection and auto-intoxication.*
19. Confusional dementia.
20. Uræmic psychosis.
21. Thyroid psychoses.
22. General paralysis.

(h). *Cerebropathies of adults.*
23. Senile dementia.
24. Traumatic insanity.
25. Cerebro-syphilitic insanity.
26. Apoplectic dementia.

**The Psychopathology of Mediums, Seers, and Mystics.**—Various observations of importance concerning the pathology of mind in mediums and professional seers have been made the subject of communication to, and discussion recently at, the Société Médico-Psychlogique of Paris.[4] Dr. Gilbert Ballet and Dr. Monier-Vinard presented a case and said that the man, who was a professional "spirit medium," presented all the characters met with in this class, namely, hyperæsthesia and proneness to develop hallucinations of the senses, especially of sight and hearing. The variety and mobility of such hallucinations were extreme. At first the medium showed slight symptoms indicating a facile tendency to "doubling of personality," which has since become more marked. "His present mental status recalls that of the visionary, Sweedenborg, the seer of planets and stars, and of life in the planets. This 'medium' seems to visit (visually by reason of his hallucinations) the planet Saturn; he sees its seas and lands, and describes in detail the appearance of the planet, the language and names of its people. He also suffers from ideas of persecution and is beginning to show signs of mental enfeeblement. The psychological difference, says Dr. Ballet, between the above patient and Sweedenborg, however, is that while the latter was a man of great intellect the patient is feeble-minded. Regarding his visit to Saturn the medium said, "Saturn is a deserted land of which the inhabitants speaked a sort of 'flayed' Greek." "His own body," he said, "was spirit; he could quit his bed and like a smoke or vapour pass into Saturn. Once there his body became grossly material again. His wife's spirit comes to him at times and makes him write automatically." Dr. Boissier[5] reported an analogous case of a medium with hallucinatory tendency and mental disequilibration. Dr. Christian[6] said he distinguished two categories of mediums, those fit to live at large because they were possessed of reason, and those who had delusions and were unfit to be at large. Their hallucinations and sensory troubles were attributed (by the mediums) to the influence of spirits, sometimes malign (Asmodeus, the devil, Ashtaroth, etc.). Hence arose the cases of demonopathy and "possession," which sometimes aroused public sympathy. Dr. Ballet concluded by saying that various degrees of spirituality, from common "visions" to automatic "spirit-writing," were present in mediums, but the one character common to all was the tendency to the dissociation of personality, and in parallelism with this was the facility for the development of visual and auditory hallucinations. As regards mystics, says Leo Gaubert,[7] respect must be paid to such sublime ethical characters as St. Theresa and Marguerite Marie. "Many saints are hysterics" is, he adds, the judgment of the closing year of the nineteenth century, which substitutes a scientific for a supernatural explanation of facts and phenomena. Saints flourished in times and surroundings when infirmity of body and mind was considered as a sign of election to grace, and the budding saint, the subjects of such infirmities, grew up in an atmosphere of highly religious nurture. Hysteria, mysticism, and demonopathy have, historically and biologically, been closely allied. Catalepsy is due to a cortical disorder of function, an insufficiency of dominance of the highest centres, a lowering of normal psychical function, and a predominance of automatism, hence the cataleptoid attitude with its plastic-rigid attitudes, its resistingness, and its proneness to automatic acts of danger or destruction. Among cataleptics, says Gaubert, were Marguerite Marie of France, a noted saint, and Lydwine of Schiedam. Medical study of her acts and symptoms by skilled mental physicians has shown that Marguerite Marie was a case of paranoia complicated with hysteria. In childhood she was inclined to become a nun; she expressed a horror of marriage, and inflicted bodily penances on herself. She entered the convent at Paray, a place then noted for its mysticism. Here she had visions of Jesus Christ, ecstacies, and cataleptiform attacks. Lydwine of Schiedam suffered from phthisis and anæmia. As a child she was fair, delicate, and of transparent complexion, with golden hair, the tuberculous offspring of tuberculous parentage. Brought up in religious superstition regarding apparitions of the Virgin and saints, thin and pale in body, strict at prayers, she developed all the faults and frailties of an invalided mind. After being long a sufferer from osteomyelitis and periosteal abscesses of chronic character and long duration, she eventually developed ascites, dropsy, and bedsores, dying a chronic, incurable invalid. Angels, said the poor deluded visionary, visited her while bedridden, and brought her cheering messages. The close of her life was marked by epileptiform convulsions and dementia, and at the age of 53 years she died. St. Theresa was born in 1515 at Axila in Spain. Early in childhood she developed, says Dr. Gaubert, religiosity and religious melancholia, and among the more definite symptoms there appeared globus hystericus, paralyses of the extremities and limbs, and later still contractures of the paralysed muscles showing clearly the grave hysteria major degenerating to organic disease. Dr. Gaubert quotes "modern instances" also of saints and seers who in his clinics and ward have exhibited similar symptoms. He concludes that Marguerite Marie had hysteria and paranoiac insanity, that Lydwine of Schiedam had scrofula and dementia, and that St. Theresa was a victim of grave hysteria.

[1] Arch. de Neur., 1904. [2] Arch. de Neur., February 1905. [3] Traité de Maladies Mentales, 1904. [4] Arch. de Neur., June 1903. [5] Ibid. [6] Ibid. [7] Thésé de Paris, 1903.

*(To be continued.)*

